# Transfer of PBMC From SSc Patients Induces Autoantibodies and Systemic Inflammation in Rag2-/-/IL2rg-/- Mice

**DOI:** 10.3389/fimmu.2021.677970

**Published:** 2021-06-23

**Authors:** Xiaoyang Yue, Frank Petersen, Yaqing Shu, Brigitte Kasper, Junie D. Tchudjin Magatsin, Marjan Ahmadi, Junping Yin, Jacqueline Wax, Xiaoqing Wang, Harald Heidecke, Peter Lamprecht, Antje Müller, Xinhua Yu, Gabriela Riemekasten

**Affiliations:** ^1^ Priority Area Asthma & Allergy, Research Center Borstel, Airway Research Center North (ARCN), Members of the German Center for Lung Research (DZL), Borstel, Germany; ^2^ Department of Histology and Embryology, School of Basic Medical Science, Guangxi Medical University, Guangxi, China; ^3^ Department of Neurology, The Third Affiliated Hospital of Sun Yat-Sen University, Guangzhou, China; ^4^ CellTrend Gesellschaft mit beschränkter Haftung (GmbH), Im Biotechnologiepark, Luckenwalde, Germany; ^5^ Department of Rheumatology, University of Lübeck, Lübeck, Germany

**Keywords:** autoimmune diseases, systemic sclerosis, granulomatosis with polyangiitis, peripheral blood mononuclear cells, autoantibodies, systemic inflammation, B cells, humanized mouse model

## Abstract

**Objective:**

The contribution of sustained autologous autoantibody production by B cells to the pathogenesis of systemic sclerosis (SSc) and granulomatosis with polyangiitis (GPA) is not fully understood. To investigate this, a humanized mouse model was generated by transferring patient-derived peripheral blood mononuclear cells (PBMC) into immunocompromised mice.

**Methods:**

PBMC derived from patients with SSc and GPA as well as healthy controls (HD) were isolated, characterized by flow cytometry, and infused into *Rag2^-/-^/IL2rg^-/-^* mice. In addition, PBMC from SSc patients treated with rituximab were transferred into mice. Twelve weeks later, human autoantibodies were determined in blood of the recipient mice and affected tissues were analyzed for pathological changes by histology and immunohistochemistry.

**Results:**

Mice engrafted with PBMC derived from SSc patients developed autoantibodies such as antinuclear antibodies (ANA) mimicking the pattern of the respective donors. Moreover, cellular infiltrates dominated by B cells were observed in lung, kidney and muscles of the recipient mice. By contrast, PBMC derived from HD or GPA patients survived in recipient mice after transfer, but neither human autoantibodies nor inflammatory infiltrates in tissues were detected. Furthermore, these pathological changes were absent in mice transferred with PBMC from rituximab-treated SSc patients.

**Conclusion:**

This humanized mouse model is indicative for cross-reactivity of human lymphocytes to murine autoantigens and argues for a pivotal role of B cells as well as of sustained autoimmunity in the pathogenesis of SSc. It provides a powerful tool to study interstitial lung disease and so far, under-recognized disease manifestations such as myositis and interstitial nephritis.

## Introduction

Systemic sclerosis (SSc) and granulomatosis with polyangiitis (GPA) are systemic autoimmune diseases affecting multiple organs. They are characterized by distinct disease phenotypes with respect to immunopathology, extent of organ involvement and clinical symptoms (1, 2). Although a number of studies have provided insights into the development of individual clinical symptoms, the mechanisms leading to these diseases are only partially understood. Autoantibodies are a common characteristic feature of both diseases (3, 4). In GPA, the pivotal role of anti-neutrophil cytoplasmic autoantibodies targeting proteinase-3 (PR3-ANCA) for the aberrant activation of cytokine-primed neutrophils was shown in studies *in vitro* and *in vivo* (2). This concept is further supported by the therapeutic effect of rituximab, a B cell-depleting antibody, in GPA. In SSc, the pathogenic role of autoantibodies and B cells is experimentally less substantiated. Here, functional antibodies e.g. against the angiotensin receptor type-1 have been suggested to play a role (5, 6). Improvement of clinical symptoms by autologous stem cell transplantation or by immunosuppressants such as cyclophosphamide or mofetil mycophenolate are suggestive for a pathogenic role of the adaptive immune system (7, 8). However, the contribution of B cells and the humoral immune response needs to be proven.

Animal models are powerful tools for studying the human disease. However, differences between species in many aspects, especially in the immune system, limit the translation from animal experiments to the clinic (9). Transferring human peripheral blood mononuclear cells (PBMC) into immunodeficient animals is an invaluable strategy to generate humanized models for autoimmune disorders (10). Particularly, this strategy is applicable for diseases in which human adaptive immune cells show cross-reactivity to animal autoantigens (10). *Rag2-/-/IL2rg-/-* mice are a promising tool for human PBMC transplantation. In addition to the lack of T cells, B cells and NK cells allowing survival of human immune cells, these mice do not express Tregs which potentially interfere with the human immune response in the animals (11). By using this model, we aimed to explore the contributions of human lymphocytes from SSc and GPA patients for the development of disease symptoms in *Rag2-/-/IL2rg-/-* mice *in vivo*.

## Methods

### Patients and Healthy Subjects

In total, 20 patients with SSc and three patients with GPA were enrolled at the Department of Rheumatology, University of Lübeck, Germany. All patients with SSc were clinically and serologically characterized according to the guidelines of the EUSTAR network and were classified according to standards defined by criteria of the American–European Consensus Group (12, 13). All patients with GPA fulfilled the American College of Rheumatology (ACR) criteria and the Chapel Hill Consensus Conference (CHCC) definition for GPA (14, 15). Four healthy donors (HD) were recruited from the Research Center Borstel, Germany. All volunteers agreed by written informed consent. This study was performed in accordance with the 1964 Helsinki Declaration, and the approval was obtained from the institutional ethics committee of the University of Lübeck (Az 16-199).

### Mice

Female Rag2 and IL-2 receptor gamma chain double deficient mice (C57Bl/6NTac;B10(Cg)-Rag2^tm1Fwa^Il2rg^tm1Wjl^, in short *Rag2^-/-^/IL2rg^-/-^*) were purchased from the Taconic Biosciences and housed under specified pathogen-free conditions with 12 hours light/darkness cycles at the animal facility at the Research Center Borstel. All animal studies have been reviewed and approved by the Animal Research Ethics Board of the Ministry of Energy Change, Agriculture, Environment, Nature and Digitalization, Kiel, Germany.

### Isolation of Human PBMC

Human PBMC were isolated using Sepmate-50 density gradient centrifugation kit according to the manufacturer’s instructions. Briefly, 45 ml of peripheral blood were collected from human donors and placed into 50 ml tubes containing heparin. Blood samples were diluted with equal volume of PBS containing 2% of fetal calf serum (FCS), and transferred to 50 ml Sepmate-50 tubes containing density gradient medium. After 10 minutes of centrifugation at 1200g at room temperature, the top layer of cells was collected into new 50 ml tubes. The collected cells were washed three times with PBS containing 2% FCS. Finally, the number of cells was counted and cells were re-suspended in RPMI1640 medium at a concentration of 1 x 10^8^ cells/ml for further assays.

### Transfer of Human PBMC Into Immunocompromised Mice

For the transfer of the cells, 2 x 10^7^ freshly isolated PBMC from healthy subjects, patients with GPA, and with SSc were injected intraperitoneally (i.p)in 100μl RPMI 1640 medium into each *Rag2^-/-^/IL2rg^-/-^* mouse. Mice received PBMC at an age of 8-10 weeks. Blood samples were collected 4 and 12 weeks after transfer and organs were harvested at the end of the experiment at week 12. Throughout the experiment, mice were weighted, and posture, skin lesions, fur texture, and diarrhea were examined weekly.

### Flow Cytometry

Numbers and phenotypes of human leukocytes were measured three times, first following their isolation and at two later time points in murine blood by flow cytometry (FACS). Briefly, 1 x 10^6^ cells of each sample were incubated with 100 µl of a fluorescence-conjugated antibody mixture in the dark at 4°C for 20 min. The antibody mixture for detection of human PBMC was composed of the following fluorochrome- conjugated antibodies: BV421-mouse-anti-human CD3 (UCHT1, Biolegend, USA), BV650-mouse-anti-human CD4 (2RPA-T4, Biolegend, USA), APC-mouse-anti-human CD8 (SK1, Biolegend, USA), PerCP/Cy5.5-mouse-anti-human CD20 (2H7, Biolegend, USA), and FITC-mouse-anti-human CD45 (2D1, Biolegend, USA). After incubation, cells were washed and resuspended in 200 μl of FACS buffer (PBS with 0.1% BSA), then 50 μl of 4% paraformaldehyde solution was added to fix the stained cells. The fixed samples were measured using an LSR II flow cytometer (BD, USA) and the data were analyzed employing FCSExpress software (De Novo Software, USA, version 5).

### Detection of Human IgG and Autoantibodies in Murine Sera

The amount of total human IgG in the sera of the PBMC-recipient mice were determined using ELISA. Briefly, 96-well ELISA plates (Thermo Fisher Scientific, USA) pre-coated with goat anti-human IgG (Polyclone, Jackson ImmunoResearch, USA) were incubated with series of diluted standard human IgG and serum samples of the mice. After washing, the plate was incubated with HRP-conjugated goat anti-human IgG antibody (Polyclone, Jackson ImmunoResearch, USA) at room temperature for 1 hour, and visualized by using TMB substrate solution. OD values were measured at 450 nm on a microreader (Tecan life science, Switzerland). The concentration (µg/ml) of human IgG in mouse sera was calculated according to the plotted standard curve. Human IgG against AT1R or ETAR in the recipient mice were detected using commercial ELISA Kits (CellTrend, Germany) according to the manufacturer’s instructions with slight modifications. In brief, plates coated with membrane extract from CHO cells overexpressing human AT1R or human ETAR (CellTrend, Germany) were incubated with serum samples diluted at 1:100. After incubation with HRP-conjugated goat anti-human IgG antibody at room temperature for 1 hour, the signal was visualized by using TMB. OD values were measured at 450 nm on a microreader (Tecan life science, Switzerland). Antinuclear antibodies (ANA) pattern detection was performed using (HEp-2) cell-based immunofluorescence staining (EUROPattern Suite, Euroimmun, Germany).

### Histological Assessment

Murine organ samples comprising spleen, skin, lung, kidney, heart, muscle and esophagus were fixed in 4% formalin overnight, embedded in paraffin, and used for preparing 3 µm–thick sections. To evaluate inflammatory and fibrotic features of the samples, paraffin sections were subjected to hematoxylin and eosin (H&E) staining (Roth, Germany) and Masson’s Trichrome staining (Sigma-Aldrich, USA), respectively. Scoring of inflammation was performed in blinded manner by two investigators. The inflammatory score was calculated based on affected area compared to total area of the tissue sample. The severity of lung fibrosis was scored according to the Ashcroft scoring system (16). For other organs, fibrosis was scored on a scale of 0 to 5: grade 0, no sign of fibrosis; grade 1, minimal fibrosis; grade 2, minimal to moderate fibrosis; grade 3, moderate fibrosis; grade 4, moderate to severe fibrosis; grade 5, severe fibrosis.

### Immunohistochemistry

Immunohistochemistry staining was performed using paraffin sections of murine tissue samples, including skin, lung, kidney, heart, muscle and esophagus. After tissue sections were deparaffinized in xylol and rehydrated in a gradient of ethanol, antigen retrieval was performed by subjecting slides to heating in 10 mM citrate buffer (pH 6.0) for 50 min. Endogenous peroxidase and unspecific binding were blocked with 3% H_2_O_2_ and 5% BSA solution. Then sections were incubated overnight at 4°C with primary antibodies recognizing CD4 (EPR6855, Abcam, Hongkong), CD20 (L26, Dako, USA) and CD138 (MI15, Biolegend, USA). Incubation with a biotinylated secondary antibody (Polyclone, Jackson Immunoresearch, USA) was conducted for 45 minutes at room temperature, followed by incubation with avidin biotinylated-HRP solution (Vector, USA) for 20 minutes. Diaminobenzidine (Vector Laboratories, USA) was applied to visualize immunoreactivity. After the sections were counter-stained with hematoxylin for 5 minutes, photos of sections were acquired using bright field microscopy (Nikon, Japan).

### Statistical Analysis

All data are expressed as mean ± SD. For quantitative data with normal distribution, statistical analysis was performed by applying a two-tailed unpaired Student’s t test or one-way ANOVA test. Fisher`s test was used to assess the significance of qualitative data. P < 0.05 was considered as statistically significant.

## Results

### Transfer and Survival of Human PBMC in Recipient Mice

**Figure 1:**
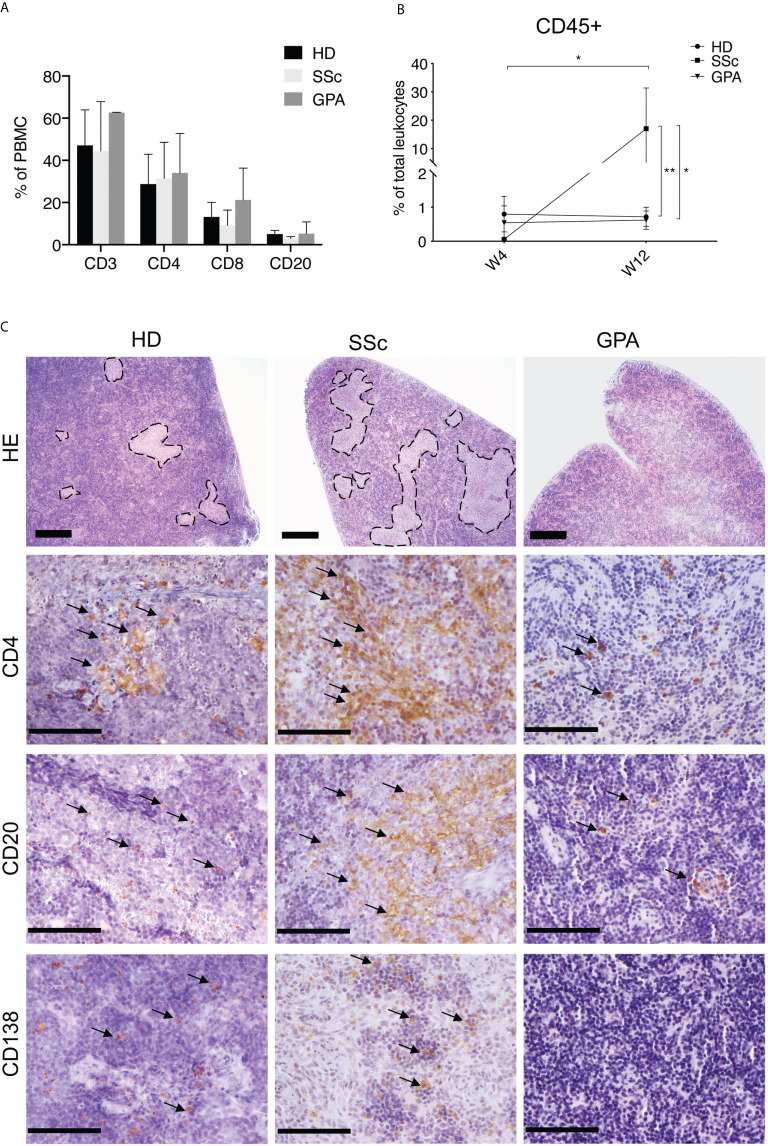
Composition and location of human PBMC in recipient mice. **(A)** Relative amounts of various lymphocyte subsets in PBMC of patients and healthy donors. The percentages of CD4+, CD8+ and CD20+ cells were detected by FACS after isolation of PBMC from healthy donors (HD, n = 5), SSc patients (n = 4), and patients with GPA (n = 3). **(B)** Relative amounts of human leukocytes detected in peripheral blood of recipient mice. Human leukocytes were quantified in murine blood 4 weeks (W4) and 12 weeks (W12) after transfer by CD45 expression using flow cytometry. The levels of human leukocytes were defined as number of human leukocytes divided by the respective total leukocyte number in murine blood (HD: n = 8, SSc: n = 5, GPA: n = 5) and expressed as percentages. **(C)** Representative micrographs of H&E stainings (bar = 500µm) and immunohistochemical stainings of human CD4^+^ T cells, human CD20^+^ B cells and human CD138^+^ cells (bar = 100 µm) in splenic sections of mice receiving PBMC from HD, SSc and GPA patients. The splenic samples were collected 12 weeks after transfer of human PBMC and prepared for histological evaluation. All data are presented as mean ± SD. Statistical analyses were performed using ANOVA (*p < 0.05, **p < 0.01).

The demographic and clinical characteristics of patients (SSc, n=9; GPA, n=3), and HD (n=) being recruited as PBMC donors are summarized (**Table 1** and **Supplementary Table 1**). Initially, PBMC derived from patients with SSc (n=6), patients with GPA (n=3) and from HD (n=) were transferred into 8, 6 and 9 *Rag2^-/-^/IL2rg^-/-^* mice, respectively. Detailed numbers of recipient mice for each donor of PBMC are given (**Table 1** and **Supplementary Table 1**). Before transfer, the cellular composition of the PBMC was detected by flow cytometry (**Figure 1A** and **Supplementary Figure 1**) and showed no major differences between the groups for the numbers of CD3^+^, CD4^+^, and CD8^+^ T cell subsets as well as CD20^+^ B cells.

**Table 1:** Demographic and clinical features of healthy donors and SSc patients.

	Donor	Health donors					Patients with SSc								
		HD_1	HD_2	HD_3	HD_4	HD_5	J_P3	J_P5	J_P6	M_P8	J_P1	J_P2	J_P8	J_P9	M_P5
Age		49	55	30	24	63	67	48	51	67	76	56	69	34	76
Gender		F	M	F	F	F	M	F	F	M	F	F	M	F	F
Subtype							Dc	Lc	Lc	lc	Dc	lc	Dc	Dc	dc
Auto-ab	ANA						**-**	**+**	**+**	**+**	**+**	**+**	**-**	**+**	**+**
	ATA						**-**	**-**	**-**	**-**	**+**	**-**	**-**	**-**	**+**
	ACA						**-**	**+**	**+**	**-**	**-**	**+**	**-**	**-**	**-**
	U1RNP						**+**	**-**	**-**	**-**	**-**	**-**	**-**	**-**	**-**
	Anti-PM_Scl							**-**	**-**	**-**	**+**	**-**	**-**	**-**	**-**
	PL-12										**+**				
	Anti-AT1R						**-**	**-**	**+**	**-**	**-**	**-**	**-**	**-**	**-**
	Anti-ETAR						**-**	**-**	**+**	**-**	**+**	**-**	**-**	**-**	**-**
Symptoms	*Raynaud						**+**	**+**	**+**	**+**	**+**	**+**	**+**	**+**	**+**
	Fatigue						**-**	**-**	**-**	**-**	**-**	**-**	**-**	**-**	**+**
	Lung fibrosis						**+**	**+**	**-**	**+**	**+**	**-**	**+**	**-**	**+**
	PAH						**-**	**-**	**-**	**-**	**+**	**+**	**-**	**-**	**+**
	Renal involvement						**+**	**+**	**-**	**(+)**	**-**	**-**	**-**	**-**	**+**
	Cardiac involvement						**(+)**	**-**	**(+)**	**+**	**+**	**-**	**+**	**+**	**+**
	Calcinoses						**+**	**+**	**+**	**-**	**-**	**-**	**-**	**+**	**+**
	Arthritis						**+**	**-**	**+**	**-**	**-**	**-**	**+**	**-**	**-**
	Sicca						**+**	**-**	**-**	**-**	**-**	**+**	**-**	**-**	**-**
	Muscle disease						**+**	**+**	**(+)**	**-**	**+**	**+**	**+**	**+**	**+**
	Digital ulcer						**+**	**+**	**-**	**-**	**+**	**-**	**+**	**+**	**+**
	Telangiectasias						**-**	**+**	**+**	**+**	**-**	**-**	**-**		**-**
	Esophagus involvement						**-**	**+**	**+**	**-**	**+**	**-**	**-**	**+**	**+**
Treatment	RTX						**-**	**-**	**-**	**-**	**-**	**-**	**+**	**+**	**+**
	Prednisolone						**+**	**+**	**-**	**-**	**-**	**+**	**+**	**+**	**-**
	MTX						**-**	**-**	**-**	**-**	**+**	**+**	**-**	**+**	**+**
	M.M.						**-**	**-**	**-**	**-**	**-**	**-**	**-**	**-**	**-**
	Ciclosporin						**-**	**-**	**-**	**-**	**-**	**-**	**-**	**+**	**-**
	HCQ						**-**	**+**	**+**	**-**	**-**	**+**	**-**	**-**	**-**
	Bosentan						**+**	**+**	**-**	**-**	**-**	**-**	**-**	**-**	**-**
	Metoprolol						**-**	**-**	**-**	**+**	**-**	**-**	**-**	**-**	**-**
	Ramipril						**-**	**-**	**-**	**+**	**-**	**-**	**-**	**-**	**-**
	Nitrendipine						**-**	**-**	**-**	**-**	**-**	**-**	**-**	**+**	**-**
	Vitamin D						**-**	**-**	**-**	**-**	**-**	**-**	**-**	**-**	**-**
	Ilomedin						**-**	**-**	**+**	**-**	**+**	**-**	**+**	**+**	**-**
No. of recipient mice		2	3	2	1	1	1	1	1	1	2	1	4	1	1

SSc, systemic sclerosis; M, male; F, female; lc, limited cutaneous SSc; dc, diffuse cutaneous SSc; ANA, anti-nuclear antibodies; ACA, anti-centromere-specific antibodies; AMA, anti-mitochondrial antibody; anti-NOR-90, anti- a 90-kDa component of the nucleolus-organizing region of chromatin; AT1R, angiotensin-II type1 receptor; ETAR, endothelin-1 type A receptor; PAH, pulmonary arterial hypertension; MTX, Methotrexate; RTX, Rituximab; M.M., Mycophenolate-Mofetil; HCQ, Hydroxychloroquine; n.a., not applicable/not available.

*Symptoms are cumulative (past & present) manifestations.

Since acute graft-versus-host disease (GVHD) is often observed in mice engrafted with human PBMC (17, 18), we monitored the body weight loss and mortality which are hallmark of GVHD during the whole experimental period. However, no body weight loss was detected in any group of mice (**Supplementary Figure 2**). In addition, other evidence of GVHD including hunching posture, skin lesions, dull fur, and diarrhea were also not observed in these mice. One of 9 mice engrafted with PBMC from HD died 2 weeks after the cell transfer, no tissue or blood samples was collected from this mouse. One of 6 mice engrafted with PBMC from GPA patients died 4 weeks after the cell transfer, and 4 of 8 mice received PBMC from SSc patients died 5, 8, 11 and 11 weeks after cell transfer, respectively. Spleen, skin, lung tissues of all these mice as well as the kidney and blood samples of part of these mice were collected from for further evaluation (**Supplementary Figure 2**).

After adoptive transfer, the engrafted human PBMC were determined in the peripheral blood of the recipient mice after four and twelve weeks by flow cytometry. Human CD45^+^ leukocytes were found in peripheral blood of recipient mice at both time points. Following transfer of PBMC, significantly increased levels of human leukocytes were detected at week 12 when compared to week four in PBMC from SSc patients (**Figure 1B**), this increase was not observed after transfer of PBMC from HD or GPA patients (**Figure 1B**). Histologically, both CD4^+^ T cells and CD20^+^ B cells migrated into the spleens of all recipient mice. CD138^+^ plasma cells were detected in the mice engrafted with PBMC from HD and SSc patients, but rarely in mice treated with PBMC from GPA patients (**Figure 1C**). Of note, transfer of PBMC from HD and SSc patients, but not GPA patients, restored the splenic white pulp in the immunodeficient mice, suggesting that human leukocytes formed germinal center-like structures (**Figure 1C**).

### Production of Human IgG and Autoantibodies by Recipient Mice

**Figure 2:**
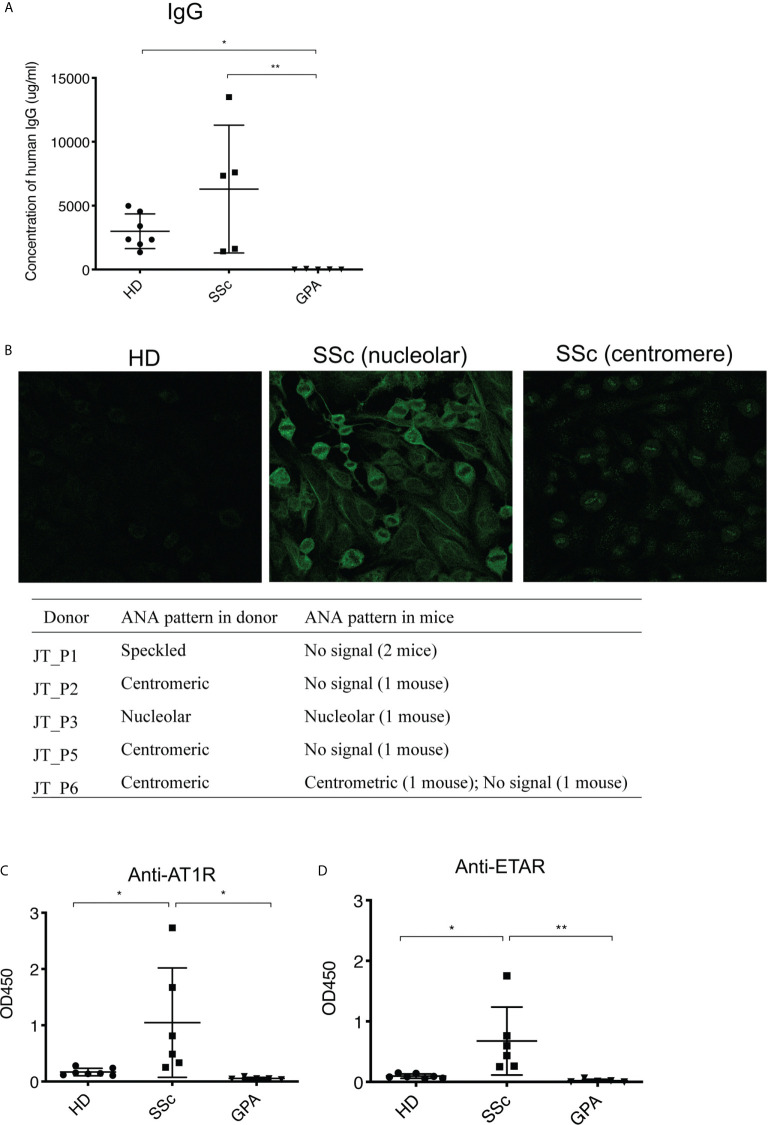
Survival and functionality of human PBMC in recipient mice. **(A)** Production of total human IgG in sera of recipient mice. The amount of total human IgG was quantified in a sandwich ELISA using human IgG-specific antibodies for capture and detection. **(B)** ANA production in sera of recipient mice. The ANA binding patterns of the mice were detected using immunofluorescence staining on human HEp-2 cells. **(C)** Levels of human IgG autoantibodies against AT1R **(C)** and ETAR **(D)** in sera of recipient mice. The autoantibodies were detected by ELISA coated with membrane extracts from CHO cells overexpressing hAT1R or hETAR. All data are presented as mean ± SD and statistical analysis was performed using ANOVA (*p < 0.05, **p < 0.01).

Given that infused human PBMC survived and formed splenic white pulp in recipient mice, the production of human IgG was evaluated as a measure of functional activity. Indeed, human IgG was detected in sera of mice transferred with PBMC from HD and SSc patients and the concentrations were comparable in both groups (**Figure 2A**). Unexpectedly, virtually no human IgG was found in sera of mice which received PBMC from GPA patients (**Figure 2A**). Since autoantibodies area major hallmark of SSc, patterns and amounts of SSc-related autoantibodies were examined in recipient mice. ANA patterns were investigated by immunofluorescence staining of HEp-2 cells. Interestingly, sera of all mice transferred with PBMC derived from HD were negative for ANA, whereas ANA were detected in sera of 2 out of 6 mice which received SSc-PBMC (**Figure 2B**). Noteworthy, the ANA patterns from these two mice were consistent with those of the corresponding SSc donors, demonstrating the recapitulation of donor ANA status in part of recipient mice. Since our previous studies demonstrated high amounts of autoantibodies targeting AT1R and ETAR in SSc and associations with clinical features, serum levels of these two autoantibodies were analyzed in PBMC-transferred mice (5). Interestingly, IgG autoantibodies against AT1R were detected in murine sera and significantly elevated in serum of mice which received PBMC taken from SSc patients in comparison to mice which received PBMC derived from HD or GPA patients (**Figures 2C, D**).

### Transfer of SSc-PBMC Induced an Infiltration of Human B Cells in Various Tissues of Recipient Mice

**Figure 3:**
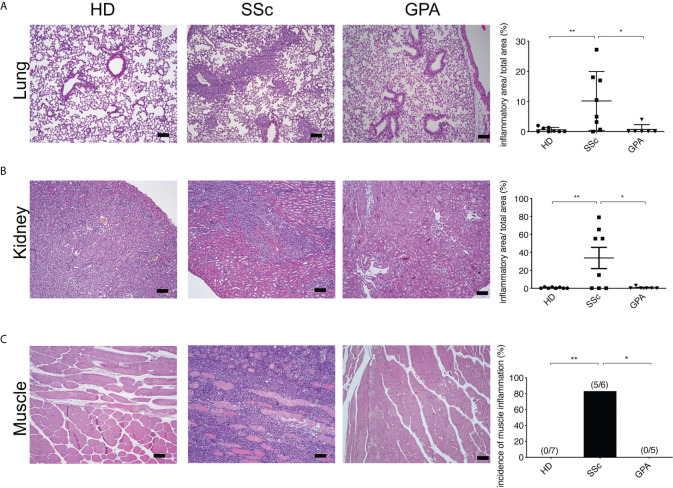
Systemic inflammation in PBMC-transferred mice. Inflammatoin in lung **(A)**, kidney **(B)**, and muscle **(C)** was kidney and muscle was evaluated by H&E staining in mice transferred with PBMC from healthy donors (HD), SSc patients and GPA patients. Representative histologic images are shown (bar = 100µm) in the left panel, while the right panel shows the quantitative score of inflammation evaluated in a blinded manner based on the ratio of infiltrated area to total area of the organ. The data are presented as mean ± SD and statistical analysis was performed using ANOVA (*p < 0.05, **p < 0.01).

To identify histopathological features typical of SSc such as inflammation and fibrosis, multiple organs including skin, lung, kidney, heart, esophagus and muscle of the recipient mice were evaluated by histology. From the mice engrafted with SSc-PBMC, 7 out of 8 animals developed pulmonary inflammation with perivascular, parabronchial as well as intra-alveolar infiltrates (**Figure 3**). Prominent inflammatory infiltrates were also observed in the kidneys (5 out of 8 animals). Moreover, muscle tissue was affected in 5 out of 6 mice engrafted with SSc-PBMC with distinct infiltrates around muscle bundles. In contrast, such signs of systemic inflammation were observed rarely in mice which received PBMC from HD or GPA patients (**Figure 3**). Additionally, mice receiving PBMC from SSc patients (n=5) developed tumors at their hind legs, which were detected as muscular masses. This was not observed in mice receiving PBMC from HD and GPA patients. No further clinical signs were observed. Overall, the body weight of the mice did not differ significantly among groups.

**Figure 4:**
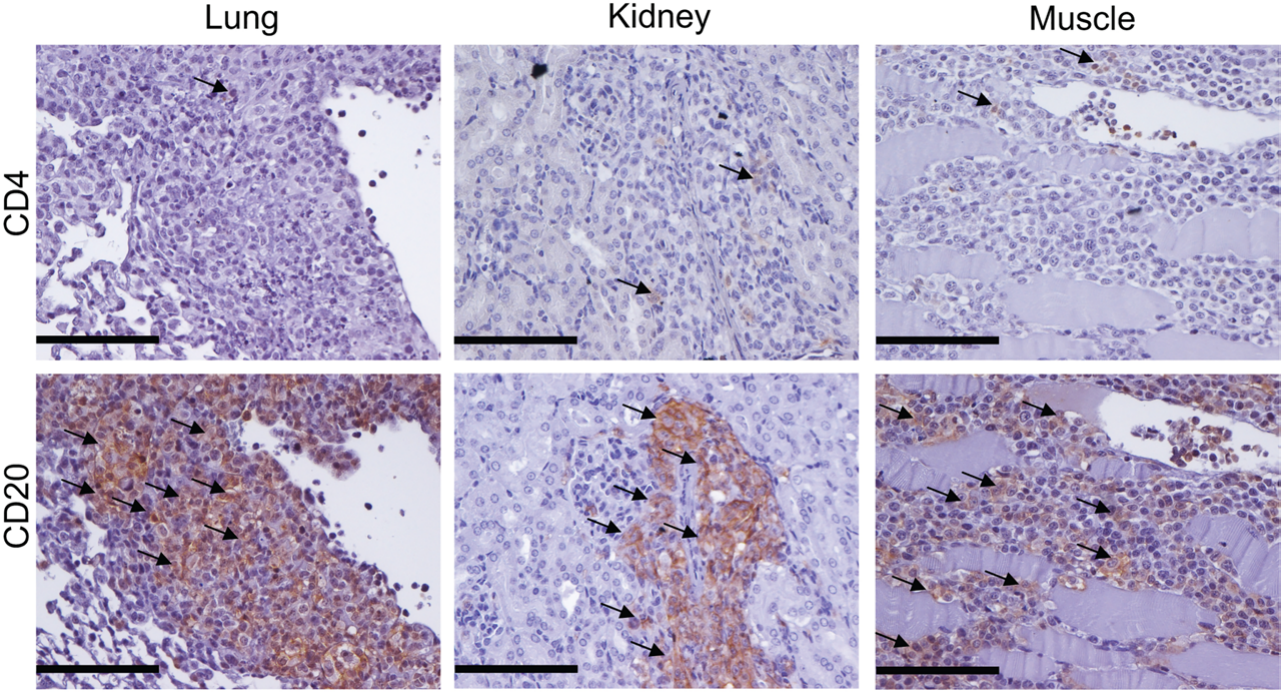
Cellular infiltrates in mice transferred with SSc-PBMC are dominated by human B cells. Human lymphocytes in lung, kidney and muscle of recipient mice were examined by immunohistochemistry staining with anti-hCD4 and anti-hCD20 antibodies. Representative micrographs of the staining are shown (bar = 100µm).

In order to characterize inflammatory symptoms in mice infused with PBMC taken from SSc patients more precisely, infiltrates were examined for the presence of human CD4^+^ T and CD20^+^ B cells by immunohistochemistry staining in various organs. Surprisingly, the majority of human cells infiltrating lung, kidney and muscles of mice treated with SSc-PBMC were CD20^+^ B cells (**Figure 4**).

To evaluate similarities and differences between manifestations of human disease and the mouse model, patient’s symptoms and manifestations were compared to those of recipient mice. Disease symptoms were often observed in lung and skin of patients, followed by muscle, kidney, esophagus and heart, while the humanized mouse model showed disease manifestation in the lungs, kidneys, and muscles (**Supplementary Table 2**). Unexpectedly, no obvious histological changes were observed in the skin in any experimental group. In addition, all mice were free of inflammation in heart and esophagus (**Supplementary Table 2**). In spite of the severe systemic inflammation in SSc PBMC-treated mice, Masson trichrome staining revealed no evident fibrosis in any tissue of the mice (**Supplementary Figure 3**).

### Transfer of PBMC Derived From SSc Patients Treated With RTX Failed to Induce Autoimmune SSc-Like Features

**Figure 5:**
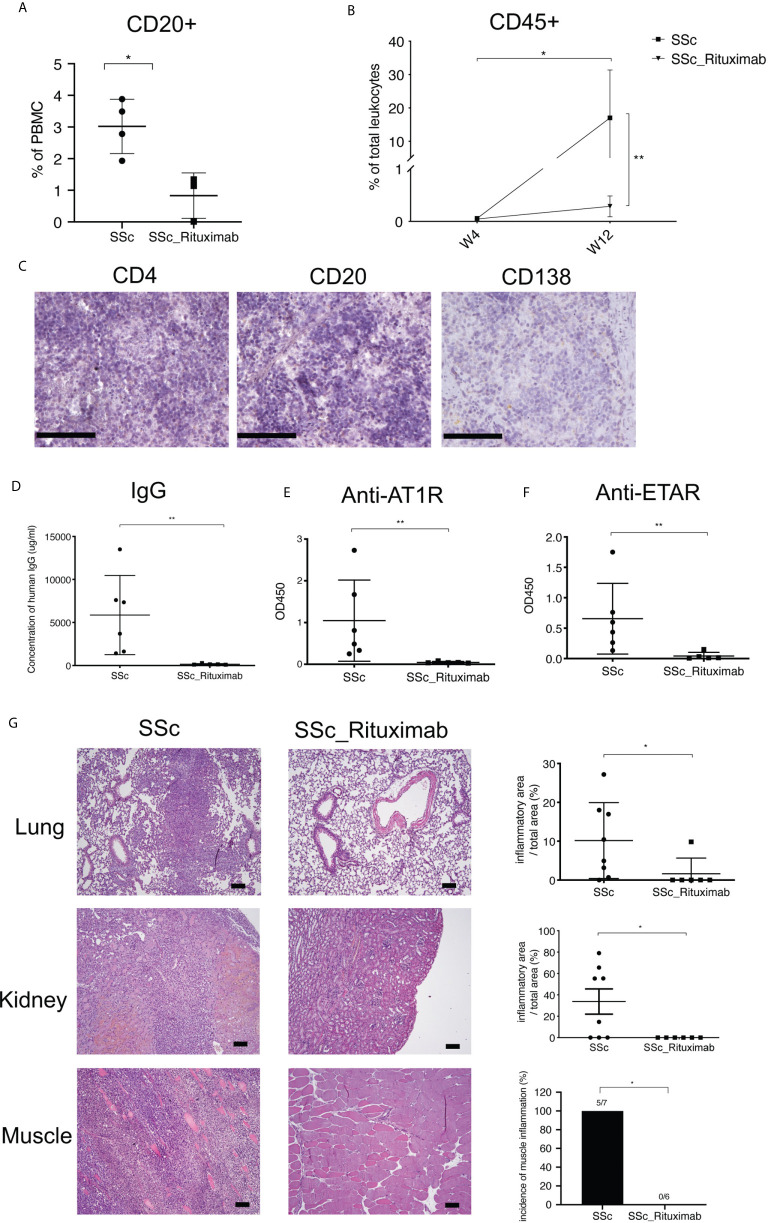
PBMC derived from Rituximab-treated SSc patients do not induce disease symptoms in mice. **(A)** Relative amounts of CD20^+^ cells in PBMC derived from untreated and Rituximab-treated SSc patients. Percentages of CD20^+^ cells in PBMC were detected by FACS after the PBMC isolation from blood samples of SSc patients (SSc) and SSc patients treated with Rituximab (SSc_Rituximab). **(B)** Relative amounts of human leukocytes in peripheral blood of recipient mice. **(C)** Representative micrographs of immunohistochemistry stainings of human CD4^+^ T cells, CD20^+^ B cells and CD138^+^ cells (bar = 100 µm) of spleens of mice receiving PBMC from SSc patients treated with Rituximab. **(D)** Production of total human IgG in sera of recipient mice. Levels of human IgG autoantibodies against AT1R **(E)** and ETAR **(F)** were determined in sera of recipient mice. **(G)** Inflammation of lung, kidney and muscle of recipient mice. Representative histologic images are shown (bar = 100µm) in the left panel, while the right panel shows the quantitative scores of inflammation evaluated in a blinded manner based on the ratio of infiltrated areas to total areas of the organ. All data are presented as mean ± SD. Statistical analyses were performed using ANOVA (*p < 0.05, **p < 0.01).

To further validate a role of B cells in the current model, 6 mice were transferred with PBMC from three SSc patients treated with rituximab (RTX). Before transfer, PBMC were characterized by flow cytometry demonstrating the ablation of B cells in the samples of RTX-treated patients (**Supplementary Figure 5**). Confirming the previous experiments, PBMC from SSc patients not receiving RTX contained significantly higher numbers of CD20^+^ B cells than those from RTX-treated SSc patients (**Figure 5A**). After transfer, numbers of human CD45^+^ peripheral blood leucocytes from RTX-treated patients were significantly lower than those observed in mice transferred with cells from SSc patients not treated with RTX (**Figure 5B**). Furthermore, the RTX-treatment also prevented the restoration of splenic white pulp in recipient mice. Here, only few CD4^+^ T cells, CD20^+^ B cells and CD138^+^ plasma cells were detected (**Figure 5C**). Accordingly, mice transferred with PBMC from RTX-treated SSc patients produced neither human IgG nor autoantibodies against AT1R or ETAR (**Figures 5D–F**). In line with the immunological findings, histological evaluations revealed no inflammation in any organs of mice receiving PBMC from RTX-treated SSc patients (**Figure 5G** and **Supplementary Table 3**).

## Discussion

In the current study, PBMC derived from SSc patients, GPA patients and HD were infused into *Rag2^-/-^/IL2rg^-/-^*mice and disease development was examined over a time period of 12 weeks. The adoptive transfer of SSc-PBMC induced the production of SSc-associated autoantibodies and signs of severe inflammation in distinct organs, thereby partially resembling inflammatory features of human SSc. To our knowledge, there is no *in vivo* evidence for direct pathogenic effects exerted by immune cells in SSc so far. Thus, our study demonstrates for the first time a sole contribution of leukocytes in mediating SSc-related features including interstitial lung disease (ILD) as the leading cause of death in SSc (19). The presence of autoantibodies and of systemic inflammation in this novel mouse model as well as the lack of these findings in mice receiving PBMC from RTX-treated SSc patients strengthen the concept of a pathogenic role of B cells and of autoantibodies as main effector molecules in SSc.

ILD, the main feature of SSc and the most frequent cause of SSc-related death, is mirrored by the current model (20). Unexpectedly, some mice treated with PBMC from SSc patients developed infiltrations in the muscles. A large proportion of SSc patients complains about myalgia, myositis or muscle weakness, but further specification requires invasive and cost-intensive assessments such as muscle biopsies, which is not done systematically (21). Noteworthy, one of the SSc patients was classified as fibromyalgia and exhibited increased myoglobin levels. The recipient mouse displayed signs of myositis. Therefore, we speculate that myositis may be an under-recognized feature of SSc. The model described here provides a novel tool for studying myositis at least in SSc and overlaps between myositis and SSc. In addition, three out of six SSc patients developed interstitial infiltrates in the kidney. In line with this, most of the patients whose PBMC induced kidney inflammation in our study exhibited a reduced glomerular filtration rate, which has not been voted as SSc-related manifestation. However, kidney involvement is common affecting 60-80% of the patients (22). Therefore, our mouse model provides an opportunity to study mechanisms of renal involvement in SSc, particularly of interstitial kidney disease. Altogether, the high percentage of mice displaying signs of myositis and interstitial nephritis shows the potential of immune cells derived from SSc patients to affect these organs. Both myopathy as well as renal disease determine quality of life and have an impact on prognosis in SSc (21–23). Differences between disease manifestations in mice and men indicate additional factors, which could affect the susceptibility and severity of a distinct manifestation.

In mice which received SSc-PBMC, infiltration of human cells was observed in lungs, muscles and kidneys. Although this is only partially known in SSc, the development of large infiltrates in the kidneys and muscles shows the potential of SSc-PBMC to affect these organs (24). Interestingly, most infiltrating human cells in mice treated with SSc-PBMC are B cells. The pathogenic role of B cells and presumably autoantibodies was clearly shown in our study by the observation that PMBC of patients treated with RTX fail to induce any disease symptoms in mice. However, the indispensable function of B cells in SSc support the concept of immunosuppressive B cell-targeted therapies in the treatment of inflammation as one central pathogenic manifestation in SSc.

To our knowledge, this is the first humanized mouse model induced by transfer of human PBMC derived from SSc patients. In our opinion, the new model offers a series of advantages compared to previous models. First, due to the application of human leukocytes this model reflects more closely the nature of human disease, and thus results derived from it may be more suited for development of therapeutic concepts. Second, by manipulating PBMC before the transfer, one can utilize this humanized model to examine the role of specific cell types. Finally, the current study demonstrates that transfer of PBMC from different patients induces different patterns of systemic inflammation, indicating that the model might be suited to develop personalized models. In the future, such models could also be used to evaluate the effects of personalized therapies.

PBMC transfer-induced humanized mouse models are always limited by acute GVHD which usually develop as early as 3-4 weeks post-engraftment (25). However, no obvious evidence of weight loss, hunching posture, diarrhea, or impaired skin integrity was observed in experimental mice used in this study during the 12-weeks experimental period (26–28). The lack of evidence of acute GVHD might be due to two major reasons. On the one hand, genetic background of recipient mice also shows strong effect on the development of GVHD. For instance, NOD mice are more susceptible to human PBMC-induced GVHD than Balb/c and C57BL/6 strains (17, 18). Since the *Rag2^-/-^/IL2rg^-/-^* mice used in this study are in C57BL/6 background, it is not surprising that these mice are relatively resistant to GVHD. On the other hand, human PBMC transfer-induced mouse model of GVHD is preferentially induced *via* i.v (29, 30). In the current study, i.p. injections were applied in accordance with some other humanized mouse models of autoimmune disorders also showing no significant GVHD (31–33). Although direct comparisons are missing, it is, therefore, conceivable that i.v. injections induce stronger GVHD than i.p. injections.

Another common limitation of PBMC transfer-induced humanized model is low engraftment rate (25). In the current study, levels of human PBMC engrafted were lower than 2% of total leukocytes in peripheral blood at week 4 after transfer, which was lower compared to previous studies (34, 35). However, this discrepancy is most likely due to the way of engraftment, where i.v. injections lead to higher circulating human PBMC in murine blood than i.p. injection. For example, recipient mice have shown an average reconstitution of 20-30% at week 4 after i.v. injection of 2-5 million PBMCs (34, 35). By contrast, in a previous study where cell engraftment was performed with i.p. injection, human cells comprised only 1.5% of the total leukocytes in SCID mice which received 15 to 30 million PBMC from patients with myasthenia gravis (36). However; although the circulating human cells are very low in the recipient mice both in the study of Martino et al. and the current study, mice developed autoantibodies and histopathological changes, indicating successful engraftment of human cells (36).

Unexpectedly, transfer of PBMC taken from patients with SSc and GPA led to substantially different outcomes in recipient mice. It needs to be mentioned that only 3 GPA patients were recruited in this study. One mouse engrafted with PBMC from a GPA patient died 4 weeks after the transfer and developed mild inflammation in the lung and kidney. No blood sample from this mouse was available for antibody test. Therefore, the conclusion on PBMC transfer from GPA in this study might be compromised due to the small number of patients studied. However, the absence of immunological and histopathological features in recipient mice might be due to a B cell dysfunction in GPA as supposed before (37). Differences in overall B cell survival and repopulation kinetics of B cell niches between GPA and connective tissues diseases have been observed following peripheral B cell depletion with rituximab (37). In GPA patients, prolonged B cell depletion was observed suggestive of a possible intrinsic defect and perturbed interaction with stromal cells (37). In addition, pathogenesis of the PBMC-transfer induced mouse model most likely depends on cross-reactivities of human autoreactive lymphocytes to murine antigens. Autoantigen(s) in SSc are highly conserved between mice and men as exemplified for DNA Topoisomerase 1, the autoantigen of ATA, with 97% homology at the protein level. In contrast, proteinase-3 (PR3), the main autoantigen in GPA, shares only 69% homology with that of mice (38). In addition, in contrast to humans, murine neutrophils express PR3 only intracellularly disabling a direct contact of potential autoantibodies with the antigen (2). Taken together, the difference between SSc-PBMC and GPA-PBMC treated mice argues that cross-reactivity of human lymphocytes to murine autoantigens and the consequent sustained autoimmunity play an essential role in the disease manifestation in PBMC transfer-induced humanized mouse models for autoimmune diseases.

In conclusion, by transferring PBMC from SSc patients into *Rag2^-/-^/IL2rg^-/-^* mice, a novel humanized mouse model was generated that resembles inflammatory mechanisms taking place particularly in early SSc. The model is focused on short-time effects and the role of immune cells infiltrating lungs, kidneys, and muscles and thus provides a novel tool to investigate partially under-recognized disease manifestations such as myositis and interstitial kidney disease. In addition, our studies highlight a pivotal role of B cells for autoimmune reactions in SSc. The humanized model provides a useful platform to study the pathogenic potential of individual immune cells *in vivo*.

## Data Availability Statement

The original contributions presented in the study are included in the article/**Supplementary Material**. Further inquiries can be directed to the corresponding authors.

## Ethics Statement

The studies involving human participants were reviewed and approved by Institutional ethics committee of the University of Lübeck. The patients/participants provided their written informed consent to participate in this study.

## Author Contributions

GR, XYu, and FP were involved in the conception and design of the study. XYue, YS, JY, JM, MA, BK, JW, and XW performed the experimental work, and collected and analyzed the data. GR, HH, PL, and AM contributed by developing and providing essential materials (blood samples of donors and ELISAs). XYu, FP, GR, and XYue were involved in drafting the manuscript. All authors contributed to the article and approved the submitted version.

## Funding

This work was supported by the Deutsche Forschungsgemeinschaft (DFG) founding the excellence cluster Precision Medicine in Inflammation, project TI4 and CD1, the RTG 1727 ‘‘Modulation of Autoimmunity’’, as well as the DFG project RI 1045 11-1-4. In addition, the mouse model was founded by the Eppenauer-Gutzeit foundation and by funding of the German Center for Lung Research (DZL).

## Conflict of Interest

HH is the owner and GR is an advisor of the company CellTrend, Germany, which produced the membrane extracts and the tests for the detection of anti-AT1R antibodies.

The remaining authors declare that the research was conducted in the absence of any commercial or financial relationships that could be construed as a potential conflict of interest.
