# Supplementary material for: Transfer of PBMC From SSc Patients Induces Autoantibodies and Systemic Inflammation in Rag2-/-/IL2rg-/- Mice
Source: Front Immunol. 2021 Jun 23;12:677970. doi: 10.3389/fimmu.2021.677970 (PMC8261241; doi:10.3389/fimmu.2021.677970)
Supplement: Supplementary file 1 [file DataSheet_1.docx]

**Supplementary table 1.** Demographic and clinical features of patients with granulomatosis with polyangiitis.

| **Patient** | **No. of recipient mice** | **Age (years)** | **Gender** | **Disease activity** | **BVAS V3.0** | **Current organ involvement** | **Autoantibody status*** | **Immunosuppressive therapy*** |
| --- | --- | --- | --- | --- | --- | --- | --- | --- |
| 1 | 2 | 59 | M | Remission | 0 | None | PR3-ANCA | None |
| 2 | 3 | 65 | M | Remission | 0 | None | PR3-ANCA | Aza |
| 3 | 1 | 73 | M | Relapse | 14 | B-symptoms, Kidney | PR3-ANCA | Pred |

GPA = granulomatosis with polyangiitis; PR3-ANCA = proteinase 3- anti-neutrophilic cytoplasmic autoantibodies; Aza = Azathioprine; BVAS V3.0 = Birmingham vasculitis activity score version 3.0; M = Male; Pred = Prednisolone. *At the time of blood collection.

**Supplementary table 2.** Organ inflammation of the mice transferred with PBMC derived from HD, SSc and GPA patients

| Group | skin | lung | heart | kidney | esophagus | intestine | muscle |
| --- | --- | --- | --- | --- | --- | --- | --- |
| HD (n=9) | 0/8 | 3/8 | 1/8 | 3/8 | 0/7 | 0/7 | 0/7 |
| SSc (n=8) | 0/6 | 7/8 | 0/7 | 5/8 | 0/7 | 0/7 | 5/6 |
| GPA (n=6) | 0/6 | 1/6 | 0/6 | 1/6 | 0/5 | 0/5 | 0/5 |

The incidence of disease in organs is expressed as the number of mice with disease / the total number of mice evaluated.

**Supplementary table 3.** Inflammation of organs of the mice receiving PBMC from Rituximab-treated SSc patients

| Group | skin | lung | heart | kidney | esophagus | intestine | muscle |
| --- | --- | --- | --- | --- | --- | --- | --- |
| SSc (n=8) | 0/6 | 7/8 | 0/7 | 5/8 | 0/7 | 0/7 | 5/6 |
| SSc B-/- (n=6) | 0/6 | 1/6 | 0/6 | 0/6 | 0/6 | 0/6 | 0/6 |

The incidence of disease in organs is expressed as number of mice with disease / the total number of mice evaluated.


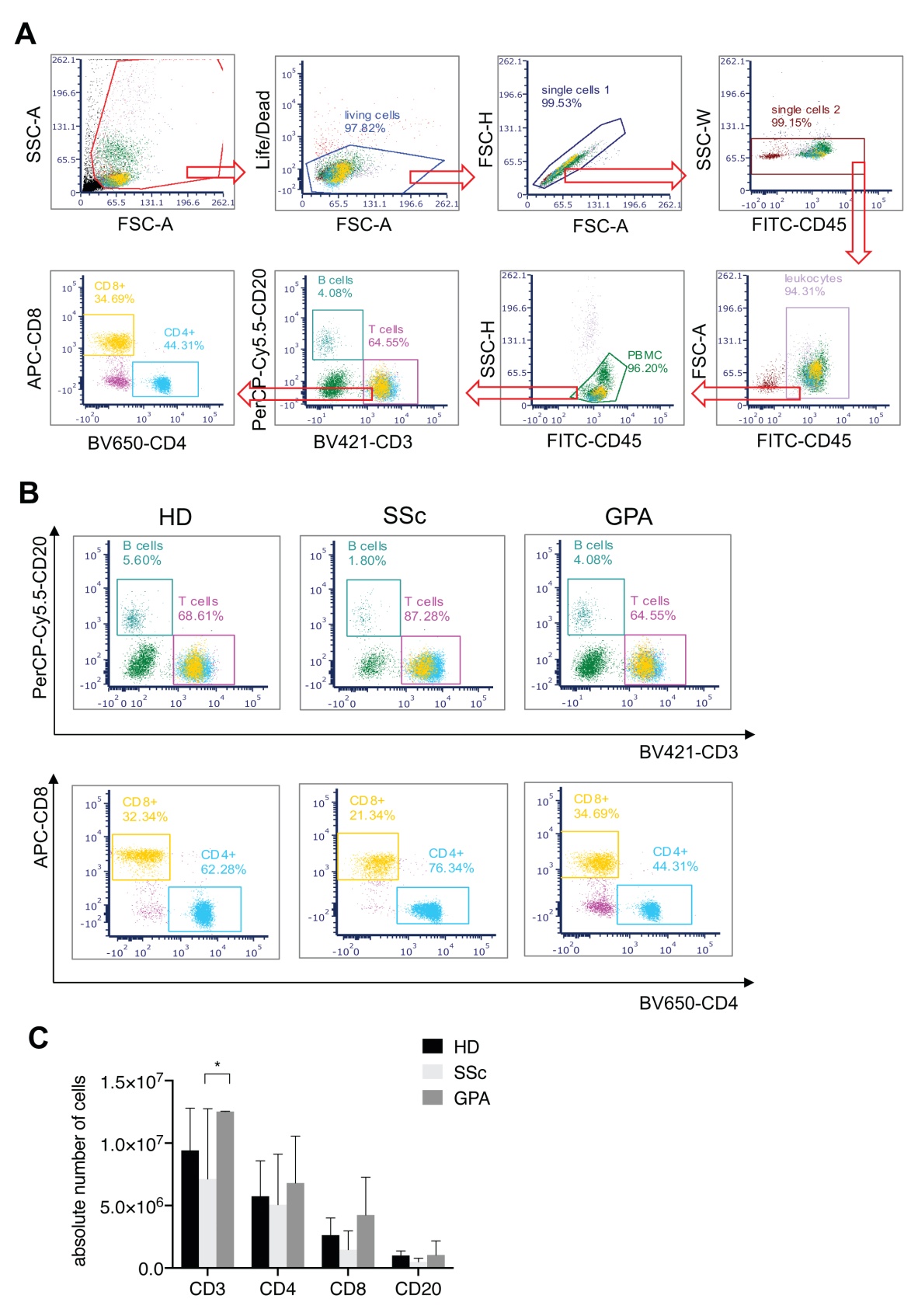


**Supplementary figure 1. Cellular compostion of PBMC from HD, SSc and GPA.** PBMC were isolated from healthy donor (HD, n=5), SSc patients (SSc, n=4) and GPA patients (GPA, n=3). Subsequently, the percentage of CD3+, CD4+, CD8+ and CD20+ cells was detected by flow cytometry. Gating strategy for flow cytometry data analyses (A) and representative FACS plots (B) for percentages of CD3+, CD4+, CD8+, and CD20+ cells in PBMC are shown. The absolute numbers of human CD3+, CD4+, CD8+, and CD20+ cells transferred to recipient mice are shown in C. The data are presented as mean ± SD. Statistical analysis was performed using ANOVA (* = p<0.05).


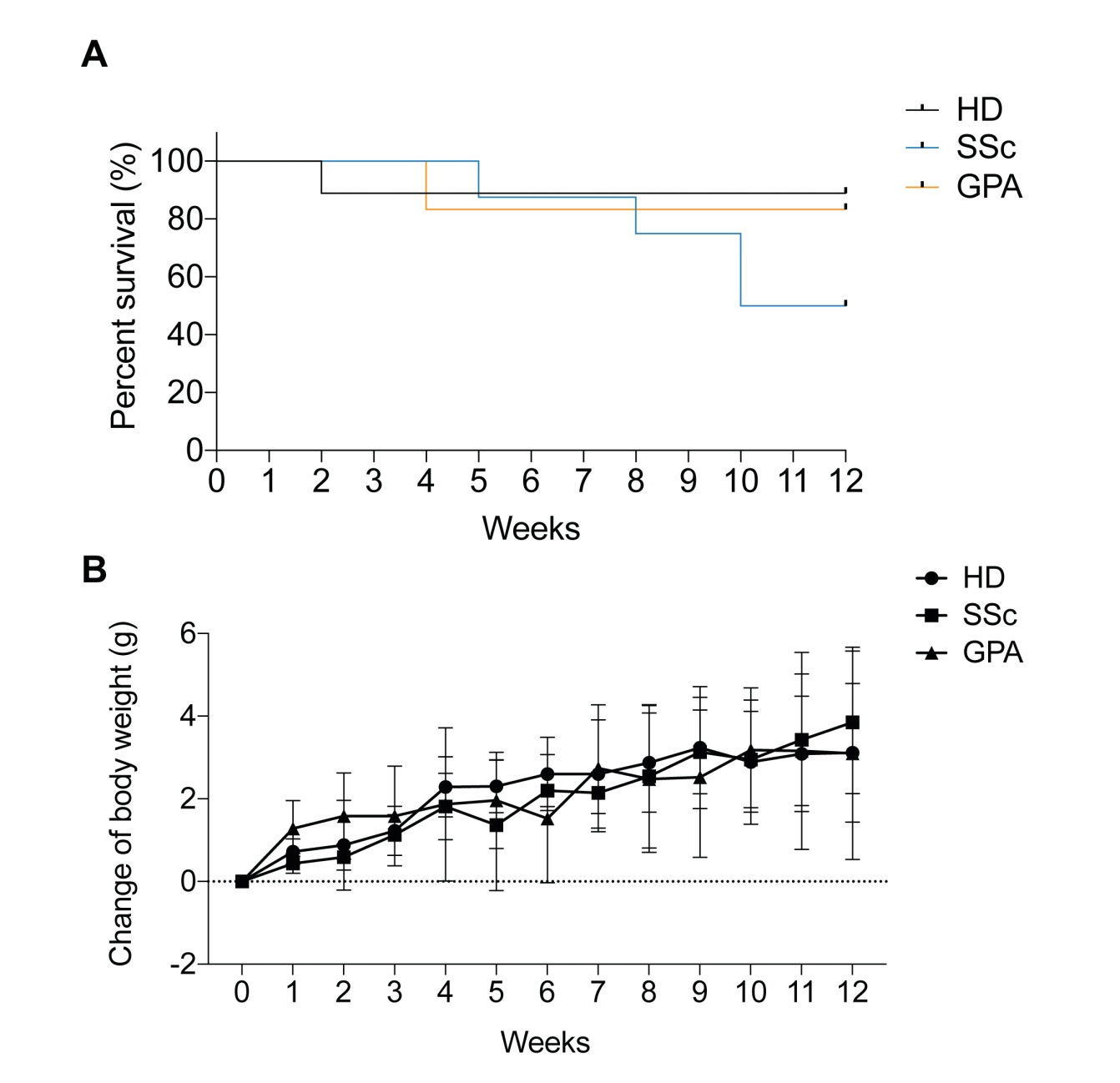


**Supplementary figure 2**. Survival and body weight of recipient mice during the experimental period. C57Bl/6NTac;B10(Cg)-Rag2^tm1Fwa^Il2rg^tm1Wjl^_,_ mice were transferred with PBMC from healthy donors (HD) (n=9), patients with SSc (n=8), or patients with GPA (n=6).Twelve weeks after the transfer, mice were sacrificed and blood and tissues were collected for further evaluation. Survival (A) and changes in body weight (B) of each mouse were monitored every week during the whole experimental period.


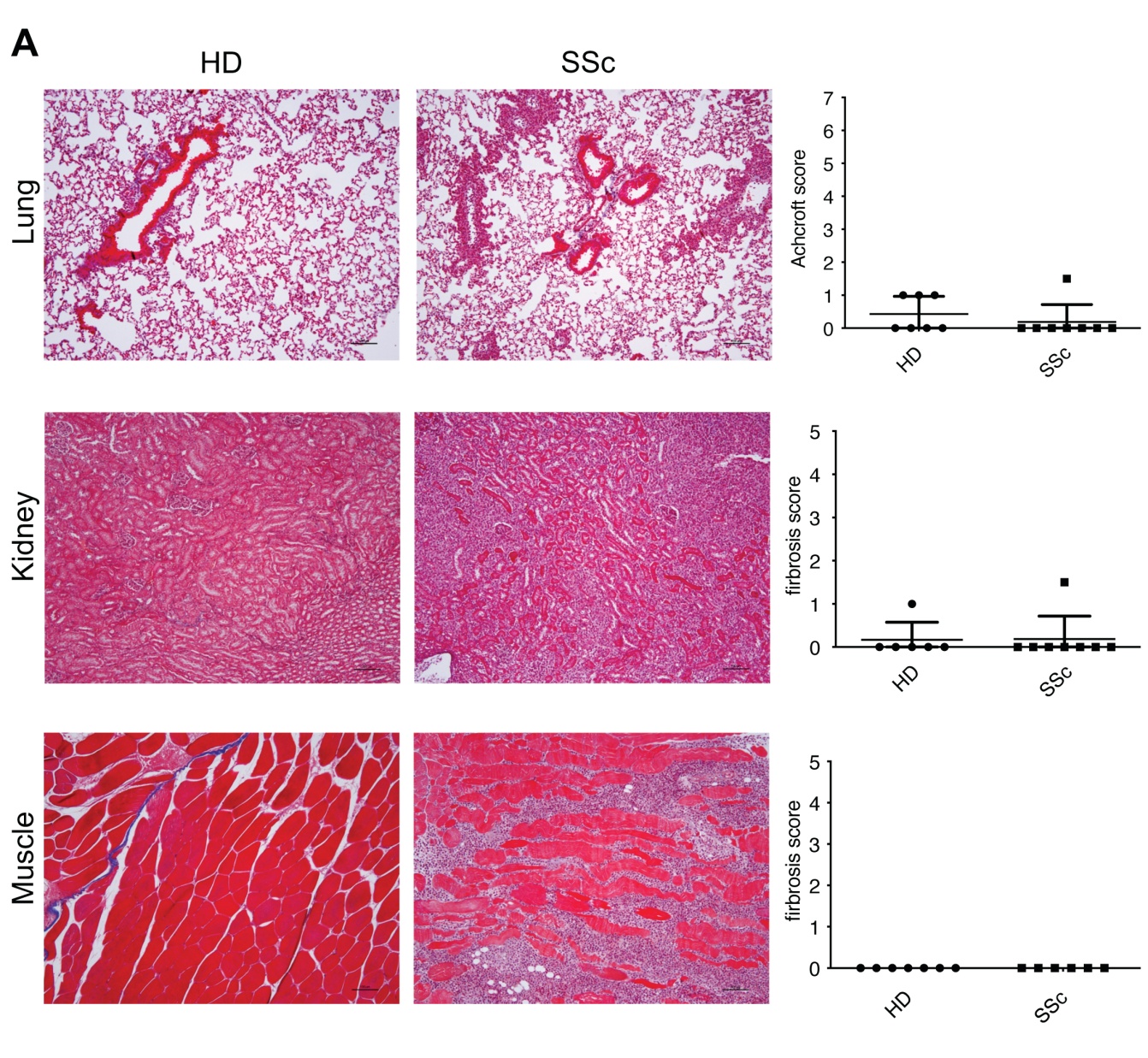


**Supplementary figure 3.** Evaluation of tissue fibrosis in mice engrafted with PBMC from HD and SSc patients. The fibrosis in lung, kidney and muscle was evaluated using Masson Thrichrome staining. Representative histological micrographs are shown (bar=100µm) in the left panel, while the right panel shows the fibrotic score evaluated in a blinded manner. The data are presented as mean ± SD. Statistical analyses were performed using Mann-Whitney test (* = p<0.05, **=p<0.01).


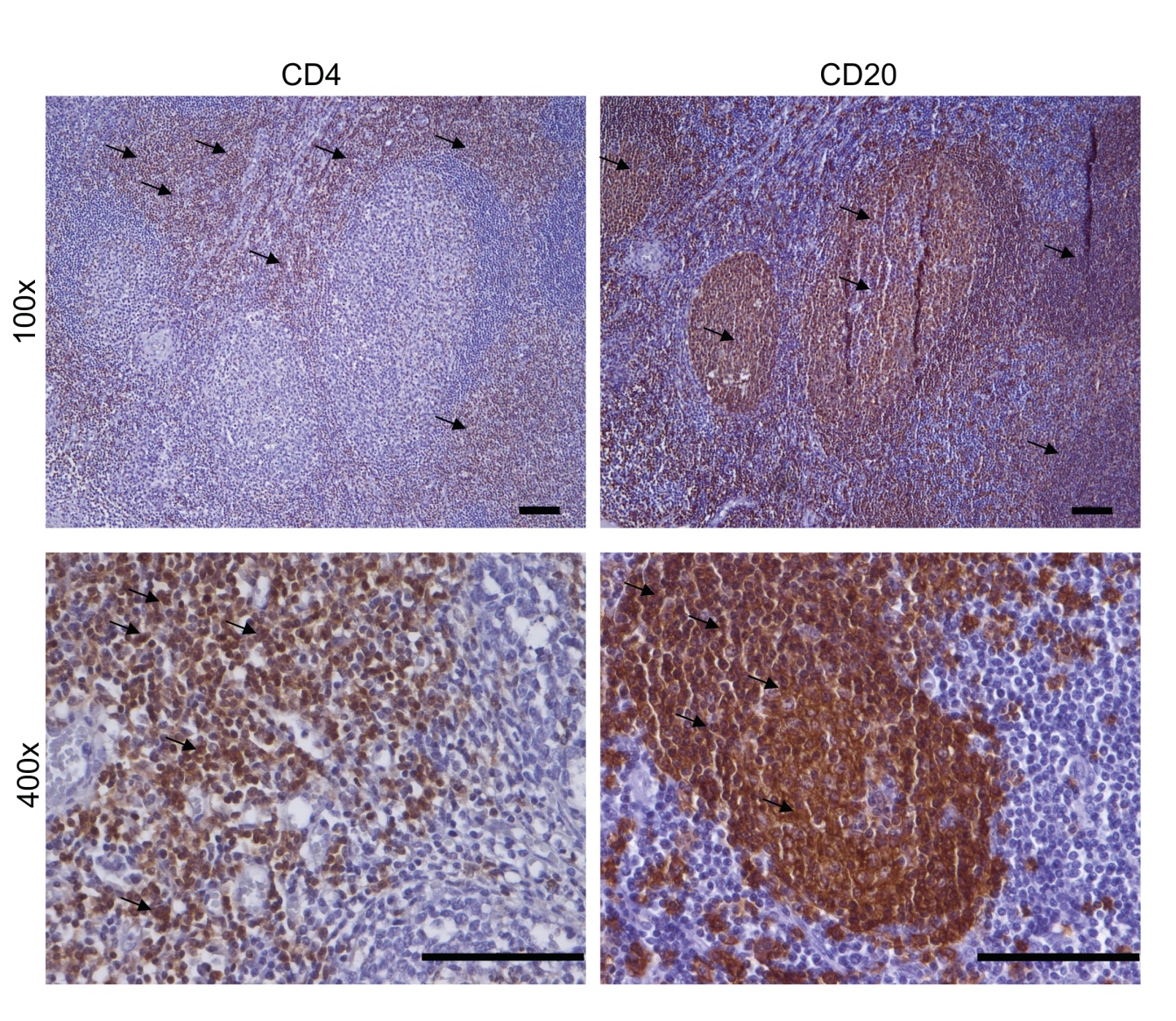


**Supplementary figure 4.** IHC staining of CD4^+^ and CD20^+^ cells in human tonsil as positive control. CD4^+^ cells (left) and CD20^+^ cells (right) in human tonsil were detected by IHC using anti-hCD4 (EPR6855, Abcam, Hongkong) and anti-hCD20 (L26, Dako, USA) antibodies. The lower (100x) and higher (400x) magnification are shown on upper and lower panel, respectively. Black arrows indicate positively stained cells. The scale bar =100μm.

**Supplementary figure 5. Relative amounts of various lymphocyte subsets in PBMC of SSc patients and rituximab-treated SSc patients.** The percentages of CD3^+^, CD4^+^, CD8^+^ and CD20^+^ cells in freshly isolated PBMC from SSc patients (n=4), and rituximab-treated SSc patients (n=3) were detected by flow cytometry.
